# The impact of physical activity on sleep architecture and cognitive function among college students

**DOI:** 10.3389/fpsyt.2025.1656278

**Published:** 2025-08-29

**Authors:** Yanbin Ji, Benhong Wang, Xiulian Sun, Zhong Wang, Wenhao Chen

**Affiliations:** ^1^ Department of Neurology, Qilu Hospital of Shandong University, Cheeloo College of Medicine, Shandong University, Jinan, China; ^2^ Department of Psychiatry, Huzhou Third Municipal Hospital, The Affiliated Hospital of Huzhou University, Zhejiang, Huzhou, China; ^3^ Department of Neurology, Research Institute of Neuromuscular and Neurodegenerative Diseases, Shandong Key Laboratory of Mitochondrial Medicine and Rare Diseases, Qilu Hospital of Shandong University, Jinan, Shandong, China; ^4^ Peking University Sixth Hospital, Peking University Institute of Mental Health, NHC Key Laboratory of Mental Health (Peking University), National Clinical Research Center for Mental Disorders (Peking University Sixth Hospital), Beijing, China; ^5^ Department of Psychiatry, The First Affiliated Hospital, Zhejiang University School of Medicine, Zhejiang Key Laboratory of Precision Psychiatry, Hangzhou, China

**Keywords:** physical activity, sleep architecture, spectral analysis, cognitive function, BDNF

## Abstract

**Background:**

Sleep constitutes approximately one-third of human life and is vital for health maintenance. Although previous studies have established an association between physical activity (PA) and sleep quality, research on the effect of PA in improving objective sleep indices remains scarce. This study investigates the impact of PA levels on sleep quality and provides direct experimental evidence to support sleep quality interventions among college students.

**Methods:**

PA and sleep parameters were assessed using sleep diaries, actigraphy, and polysomnography (PSG). The spectral analysis was performed on PSG-acquired electroencephalographic (EEG) data to examine power distribution variations across distinct sleep stages. Concurrently, venous blood samples were collected for quantitative analysis of serum brain-derived neurotrophic factor (BDNF) levels using enzyme-linked immunosorbent assay (ELISA). Cognitive functions were assessed using the Psychomotor vigilance task (PVT).

**Results:**

Actigraphy-derived 7-day average daily steps demonstrated significant positive correlations with PSG-measured sleep efficiency (SE) and non-rapid eye movement sleep stage 2 (N2), while exhibiting negative correlations with wake after sleep onset (WASO) and wake stage. The results of multiple linear regression analysis showed that with the increase in the average daily steps, SE and N2 time increased, and wake stage and WASO decreased. Spectral analysis of sleep EEG data demonstrated that average daily steps positively correlated with the mean power of slow sigma and fast sigma during NREM sleep, as well as mean theta power during REM sleep. Additionally, this study revealed that higher average daily steps correlated with elevated BDNF concentrations and reduced reaction time in the PVT task.

**Conclusions:**

This study demonstrates that there is an association between PA and the modulation of sleep architecture and cognitive function among college students. These findings gain additional support from sleep EEG microstructural analyses, with the observed improvements potentially mediated through PA-mediated modulations in circulating BDNF levels.

## Introduction

Sleep is one of the most fundamental biological processes, essential for maintaining both physical and psychological health. Adequate and high-quality sleep plays a crucial role in restoring energy and physical strength, protecting the brain, and regulating emotional states (1, 2). As a vital component of a healthy lifestyle, sleep quality has been closely linked to heart health, metabolic efficiency, cognitive function, and overall mental well-being. However, rapid socioeconomic development and resulting changes in lifestyle and work patterns over recent decades have led to a dramatic increase in sedentary behavior, particularly among younger populations such as college students. At the same time, the prevalence of insufficient or poor-quality sleep has steadily risen, raising public health concerns about its short- and long-term consequences (3, 4).

Physical activity (PA) is defined as any bodily movement produced by skeletal muscles that results in energy expenditure. It includes a wide range of activities such as occupational work, sports, exercise, and household chores (5). The strong interrelationship between PA and sleep health has been recognized in various studies; it is widely accepted that regular and moderate PA may improve sleep onset, duration, and depth (6, 7). For college students, a population facing academic stress, lifestyle irregularities, and frequent use of electronic devices, sleep disturbance and insufficient activity levels are particularly prevalent. Identifying accessible and effective strategies to promote better sleep in this group thus has significant importance.

Despite the established association between PA and sleep, there remain several critical research gaps. First, most studies have relied on subjective self-report questionnaires to evaluate sleep quality, which, although convenient, are prone to bias and may not fully capture objective changes in sleep architecture. Second, there is a lack of consensus on the most valid and user-friendly indicator of daily PA for predicting sleep outcomes in real-world settings; while measures such as calorie consumption or metabolic equivalents are commonly used, they often require complex calculations or are less intuitive for the general public. In contrast, the daily steps, an easily understood and widely accessible parameter, has been proposed as a practical metric for assessing PA in diverse populations (8–10). Higher daily steps have been associated with fewer depressive symptoms in both cross-sectional and longitudinal studies conducted among the general adult population (11). A population-based prospective cohort study further revealed that walking 10,000 steps per day might be linked to reduced mortality rates, as well as lower incidences of cancer and cardiovascular diseases (9). With the advent of portable monitoring technology, such as actigraphy, it has become possible to non-invasively and reliably track both daily movement and sleep-wake patterns (12).

The mechanisms linking PA and sleep require further exploration. Evidence indicates that evening exercise increases nocturnal non-rapid eye movement sleep (NREM) latency, particularly by enhancing slow-wave activity on the electroencephalogram (EEG) (13). This slow-wave sleep promotes bodily and neural recovery, facilitates the clearance of metabolic waste, and supports memory consolidation (14). Sleep has also been found to be involved in the regulation of neuroendocrine factors. Brain-derived neurotrophic factor (BDNF), abundantly present in brain regions critical for learning and memory, is essential for neuronal survival, differentiation, and synaptic plasticity, and also plays a key role in sleep regulation (15). Studies show that moderate aerobic exercise significantly raises BDNF levels in both the central and peripheral nervous systems, with the magnitude of this effect influenced by exercise intensity and duration. Elevated BDNF contributes to greater brain plasticity and offers protection against depression and anxiety (16, 17).

To address these gaps, the present study investigated the effect of PA, measured by daily steps, on sleep quality among college students using both subjective and objective methods. By employing actigraphy for continuous PA assessment, sleep diary recording, and polysomnography (PSG)-the gold standard for sleep measurement-as well as serum BDNF sampling across the sleep-wake cycle, this research aims to not only delineate the relationships among PA, sleep quality, cognitive function, and BDNF secretion, but also provide practical evidence for clinical interventions. By focusing on a homogeneous sample of college students and rigorously excluding individuals with clinically significant anxiety or depression, this study also seeks to minimize confounding factors and clarify the potential of increasing average daily steps as an accessible strategy for improving sleep quality.

In summary, this study aims to investigate the relationship between the amount of daily physical activity and both nighttime sleep quality and daytime functioning, as well as to explore the neural mechanisms that may mediate the associations among physical activity, sleep architecture, and cognitive function. The research results may provide information for sleep management and health promotion strategies in college campuses and other settings, and offer a scientific basis for promoting daily PA as a means to improve the sleep and overall health of young people.

## Materials and methods

### Subjects

Twenty-three college students were recruited via advertisements. All participants confirmed maintaining a stable sleep-wake cycle (bedtime: 23:00 ± 30 minutes; wake time: 07:00 ± 30 minutes) for a minimum of six consecutive months prior to study enrollment, as verified through one-week sleep diary. Exclusion criteria included: (1) current or past medical illness; (2) regular use of psychoactive substances (caffeine, alcohol, tobacco); (3) any medication within 6 months; and (4) recent shift work or trans-meridian travel (in the past 6 months). Eligible candidates were screened, including a structured clinical interview and physical examination based on DSM-IV. The study protocol (Chinese Clinical Trial Registry ID: ChiCTR-ROC-17012112) received ethical approval from Peking University Sixth Hospital’s Institutional Review Board. All participants provided written informed consent prior to enrollment. Complete demographic and baseline characteristics are presented in **Table 1**.

**Table 1:** General clinical data of subjects (n=23).

Characteristic		Sample amount	Characteristic		Sample amount
Male (%)		10 (43.47%)	BMI		21.77 ± 2.09
Age (years)		22.70 ± 2.36	HAMD		1.26 ± 1.42
Undergraduate degree		16.35 ± 1.85	HAMA		1.65 ± 1.23
Average daily steps		11085.63 ± 1786.40	ACT	TAK	2.50 ± 0.77
Sleep diary	TIB	496.24 ± 20.38	PSG	TST	446.17 ± 40.13
	TST	466.30 ± 22.80		SE (%)	89.72 ± 7.92
	SOL	19.86 ± 7.39		SOL	17.63 ± 25.41
	NAK	0.62 ± 0.64		WASO	33.39 ± 35.53
ACT	TST	428.38 ± 31.11		W	43.30 ± 37.53
	SE (%)	85.87 ± 5.36		R	81.09 ± 17.32
	SOL	8.61 ± 5.58		N1	37.85 ± 12.64
	WASO	62.59 ± 27.25		N2	208.93 ± 34.20
	NAK	24.11 ± 7.25		N3	118.30 ± 30.26

The data are expressed as the mean ± SD or numbers (%). HAMD, Hamilton depression rating scale; HAMA, Hamilton anxiety rating scale; BMI, mean body index. ACT, actigraphy; PSG, polysomnography; TIB, total time in bed; TST, total sleep time; SOL, sleep onset latency; NAK, number of awakenings; SE, sleep efficiency; WASO, wake time after sleep onset; TAK, average time of awakenings; W, wake stage; REM, rapid eye movement sleep time; N1, duration of non-rapid eye movement sleep stage 1; N2, duration of non-rapid eye movement sleep stage 2; N3, duration of non-rapid eye movement sleep stage 3.

### Experimental design

**Figure 1:**
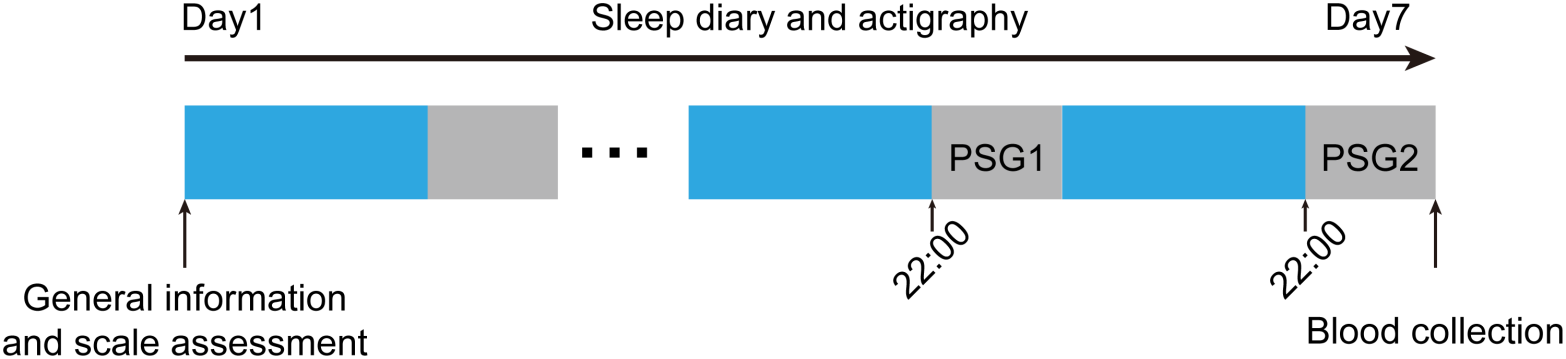
Flow chart of the study. Prior to experimental commencement, participants underwent demographic data collection and psychometric assessment. All subjects completed sleep diary and actigraphy records for 7 consecutive days. Overnight PSG was conducted on nights 6 and 7, with fasting venous blood samples collected following the final PSG monitoring. PSG, polysomnography.

Prior to the formal experiment, comprehensive demographic data collection and standardized psychometric assessments were conducted for participant screening. Qualified participants were instructed to maintain stable PA regimens and habitual sleep-wake cycles throughout the study period, with significant deviations prohibited. All subjects underwent continuous wrist-actigraphy monitoring and maintained detailed sleep diaries for seven consecutive days to establish baseline activity-rest patterns. To control for potential first-night effects and screen for sleep pathologies, initial PSG recordings were obtained on Night 6, with primary PSG data collected on Night 7 for subsequent analysis. Fasting venous blood samples were acquired immediately following the final PSG monitoring (**Figure 1**).

### Questionnaire and scale assessment

Prior to the experiment, all participants underwent a comprehensive screening process, including a detailed interview and baseline assessments such as age, gender, years of education, height, and weight. Participants provided detailed self-reported information about their daily activities and medical history, with particular attention to any mental or physical illnesses, especially sleep-related disorders. The study employed the 24-item Hamilton depression rating scale (HAMD) to evaluate the severity of depression in participants over the past week (18). Similarly, the Hamilton anxiety rating scale (HAMA) was used to assess anxiety levels during the same period (19). To minimize confounding effects, participants with HAMD scores ≥8 or HAMA scores ≥7 were excluded from the study.

### Subjective sleep assessment

Participants maintained a sleep diary for one week, documenting bedtime (lights-off time), sleep onset latency (SOL), nocturnal awakenings, morning awakening and rising times, total sleep time (TST), post-awakening subjective feelings, and additional factors such as caffeine intake, medication use, and PA levels. Total time in bed (TIB), TST, SOL, and number of awakenings (NAK) were computed to assess participants’ subjective sleep quality.

### Wrist actigraphy for PA and sleep monitoring

Actigraphy (wGT3x-BT, Actigraphy, ACT) is a device for assessing activity levels and objectively measuring PA and sleep patterns (20). Following data collection, ActiLife 6 and MATLAB software are used to process the data and generate a clinical report based on sleep diary records. The recorded data includes subject name, wearing time, daily steps, TST, sleep efficiency (SE), SOL, wake time after sleep onset (WASO), NAK, and average time of awakenings (TAK). To minimize variability in average daily steps, participants were required to wear the device for 7 consecutive days with a minimum wearing time of 2 hours per day (21). Wearing time during sleep must exceed 1 hour, otherwise, the data is deemed invalid (22). Only data meeting the criteria for at least 4 days was included in the statistical analysis.

### PSG to monitor sleep parameters and spectral-frequency analysis

The PSG recordings were performed using a Compumedics Grael series PSG device (Compumedics Sleep Study System, Melbourne, Australia). This system continuously monitored participants’ sleep architecture throughout the night, acquiring EEG signals from F3, F4, C3, C4, O1, and O2 channels, along with electrooculogram (EOG) and electromyography (EMG) signals. Simultaneously, it monitored snoring, oral/nasal airflow, and blood oxygen saturation. The recording parameters were standardized as follows: a sampling frequency of 256 Hz with high-pass and low-pass filters set at 0.3 Hz and 35 Hz, respectively. Prior to monitoring, electrode impedances were verified for all channels, and any location exceeding 5 kΩ was repositioned to meet the recording standard. PSG recordings were conducted in an environment with controlled temperature and humidity to minimize noise interference.

The PSG data were interpreted and analyzed using Compumedics Profusion PSG3 sleep analysis software. Sleep stages were scored in 30-second epochs, while limb movements and respiratory events were analyzed in 2-minute epochs. The following sleep parameters were derived and reported: TST, SE, SOL, wake stage (W), non-rapid eye movement sleep stage 1 (N1), non-rapid eye movement sleep stage 2 (N2), non-rapid eye movement sleep stage 3 (N3), rapid eye movement sleep (REM) stage and WASO.

For power spectral analysis of EEG signals, all preprocessed sleep EEG data underwent rigorous manual artifact inspection to EOG and EMG, thereby ensuring high data quality. Spectral analysis of artifact-free EEG data was performed using the Welch method (fast Fourier transform, FFT) in MATLAB (The MathWorks Inc., Natick, MA). Parameters included a 5-second Hanning window applied to consecutive non-overlapping segments of 30-second epochs with 50% overlap, providing a frequency resolution of 0.25 Hz. Average power for specific frequency bands was then calculated separately for NREM and REM stage (23). Relative power was computed for seven clinically relevant frequency bands: slow oscillations (SO, 0.5-1.0 Hz), slow-wave activity (SWA, 0.5-4.0 Hz), delta (1.0-4.0 Hz), theta (4.0-8.0 Hz), slow sigma (9.0-12.0 Hz), fast sigma (12.0-15.0 Hz), and beta (16.0-30.0 Hz) (24–26).

### Enzyme-linked immunosorbent assay (ELISA)

Blood samples were collected within 30 minutes of waking on day 7. Samples were clotted at room temperature for 60 min, centrifuged at 1000 × g for 15 min, and the serum was immediately aliquoted and stored at −80°C until analysis. Serum BDNF levels were quantified using ELISA kits (DBD00, R&D Systems, Minneapolis, MN, USA).

### Psychomotor vigilance task

After the PSG recording on the second night of this study, the participants were arranged to take the test in a quiet and comfortable environment. The PVT protocol, implemented using E-Prime software (version 1.0), presented participants with a red fixation square centrally positioned on the display. Following variable interstimulus intervals (ranging from 1,000 to 9,000 ms), a millisecond counter (yellow digits) appeared, initiating from 0 ms. Participants were instructed to respond to stimulus onset as rapidly as possible via left mouse button press within the 30,000 ms response window. Successful responses immediately halted the counter and triggered visual feedback (yellow box) displaying the achieved reaction time (RT) for 2,000 ms before trial termination. The complete assessment comprised 90 trials with an approximate total duration of 10 minutes. Post-test analysis classified trials according to conventional PVT performance metrics: lapses (RT ≥ 500 ms) and false starts (RT ≤ 150 ms), with valid responses defined as 150 ms < RT < 500 ms.

### Statistical analysis

All statistical analyses were conducted using SPSS 26.0 (IBM Corp., Armonk, NY, USA) and GraphPad Prism 9.0 (GraphPad Software, San Diego, CA, USA). Continuous variables are expressed as mean ± standard deviation (SD). Normality of data distribution was assessed using Shapiro-Wilk tests. Based on the normality test results, we employed Pearson correlation analysis for normally distributed data and Spearman correlation analysis for non-normally distributed data. To evaluate the relationship between PA and sleep quality, multiple linear regression analysis was performed. Statistical significance was set at *P* < 0.05.

## Results

### Demographics and clinical characteristics of the subjects

**Table 1** presents the demographic and clinical characteristics of all participants. This study comprised 10 males and 13 females (mean age = 22.70 ± 2.36 years; mean BMI = 21.77 ± 2.09 kg/m²). All participants scored within normal ranges on depression and anxiety questionnaires. Actigraphy data revealed average daily steps of 11,085.63 ± 1,786.40. The study enrollment process is illustrated in **Figure 1**.

### Correlation between average daily steps and sleep parameters by sleep diary, ACT, and PSG

**Figure 2:**
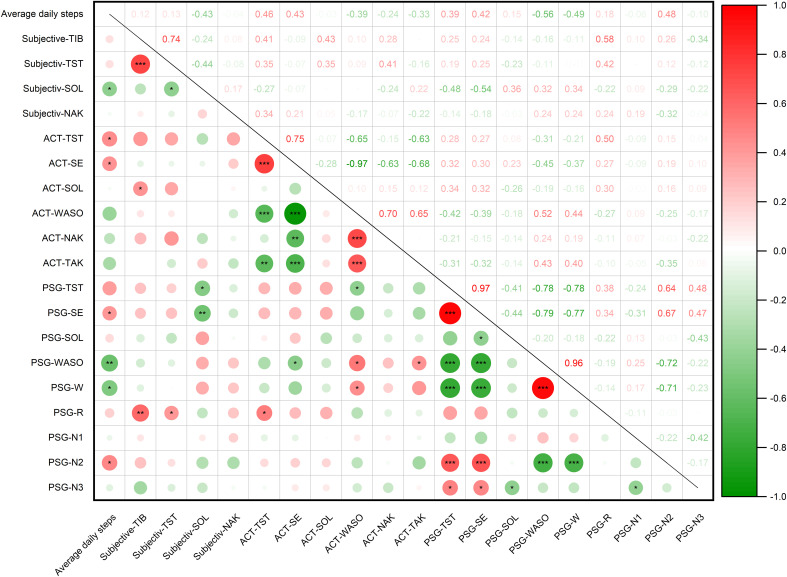
The correlation between the average daily steps and sleep parameters by sleep diary, ACT and PSG. TIB, total time in bed; TST, total sleep time; SOL, sleep onset latency; NAK, number of awakenings; SE, sleep efficiency; WASO, wake time after sleep onset; TAK, average time of awakenings; W, wake stage; REM, rapid eye movement sleep stage; N1, duration of non-rapid eye movement sleep stage 1; N2, duration of non-rapid eye movement sleep stage 2; N3, duration of non-rapid eye movement sleep stage 3. *P < 0.05, **P < 0.01, ***P < 0.001.

Subjective sleep parameters were documented via standardized sleep diary, contrasting with the objective physiological measurements obtained through ACT and PSG. The correlation analyses were employed to investigate potential covariation between habitual PA (operationalized as average daily steps) and these distinct sleep assessment paradigms. The results showed that the average daily steps were significantly associated with the SOL (*r* = -0.43, *P* = 0.039), but no significant association was observed with TIB, TST, or NAK in sleep diary (**Figure 2**). ACT data revealed that average daily steps exhibited a positive correlation with TST (*r* = 0.46, *P* = 0.028) and SE (*r* = 0.42, *P* = 0.044), but no significant correlation with SOL, WASO, NAK, or TAK (**Figure 2**). Additionally, our study demonstrated that average daily steps were positively associated with SE (*r* = 0.42, *P* = 0.048), and N2 stage (*r* = 0.48, *P* = 0.021), and inversely associated with WASO (*r* = -0.56, *P* = 0.005) and wake stage (*r* = -0.49, *P* = 0.018). Notably, no significant correlation was observed with TST, SOL, REM, N1, or N3 stage in the PSG data (**Figure 2**). To comprehensively investigate the relationship between PA and sleep quality among college students, we conducted a multivariate linear regression analysis using stepwise entry method. The model incorporated gender, age, education level, BMI, HAMD and HAMA scores as covariates, with average daily steps (measured in 1000-step increments) as the primary predictor and PSG-derived sleep parameters as dependent variables. Our regression analysis revealed the subjects increased average of 1,000 steps per day, and the sleep parameters SE and N2 stage increased by 1.85% and 9.16 minutes, and WASO and wake stage decreased by 11.34 minutes and 10.35 minutes recorded by PSG, respectively (**Table 2**). Our data show that increased PA may enhance sleep quality by both prolonging restorative N2 stage and reducing nocturnal awakenings. Notably, this study reveals measurable discrepancies between subjective self-reported sleep data and objective sleep measurements, highlighting the importance of differentiating these assessment methods when evaluating sleep quality.

**Table 2:** Multiple linear regression analysis of average daily steps and sleep parameters by PSG.

Variable	*B*	SEM	*β*	*t*	*P*	*R^2^*
SE	1.85	0.98	0.42	2.10	0.048^*^	0.17
WASO	-11.34	3,61	-0.56	-3.13	0.005^**^	0.32
W	-10.35	4.02	-0.49	-2.56	0.018^*^	0.24
N2	9.16	3.67	0.48	2.50	0.021^*^	0.23

SE, sleep efficiency; WASO, wake time after sleep onset; W, wake stage; N2, duration of non-rapid eye movement sleep stage 2. *P < 0.05, **P < 0.01.

### Correlations between average daily steps and EEG spectral power

Spearman correlation analysis revealed positive associations between average daily steps and the mean power of theta (*r* = 0.56, *P* = 0.006), slow sigma (*r* = 0.54, *P* = 0.007), and fast sigma (*r* = 0.43, *P* = 0.039) activity during NREM sleep, and the mean power of theta (*r* = 0.49, *P* = 0.019) during REM sleep (**Tables 3**, **4**). These findings suggest that increased average daily steps may contribute to enhanced sleep continuity, deeper sleep, and improved memory function.

**Table 3:** The correlation between the average daily steps and EEG spectral power in NREM.

	SO	SWA	Delta	Theta	Slow sigma	Fast sigma	Beta
*r*	0.09	0.18	0.19^#^	0.56	0.54	0.43	0.12
*P*	0.673	0.412	0.380	0.006^**^	0.007^**^	0.039^*^	0.590

^#^Represents Pearson correlation, and the rest are Spearman correlation. SO, slow oscillations; SWA, slow-wave activity. *P < 0.05, **P < 0.01

**Table 4:** The correlation between the average daily steps and EEG spectral power in REM.

	SO	SWA	Delta	Theta	Slow sigma	Fast sigma	Beta
*r*	0.08^#^	0.35	0.29	0.49	0.31	0.21	0.22
*P*	0.715	0.101	0.173	0.019^*^	0.157	0.335	0.312

^#^Represents Pearson correlation, and the rest are Spearman correlation. SO, slow oscillations; SWA, slow-wave activity. *P < 0.05.

### Correlation between average daily steps and cognitive function

**Figure 3:**
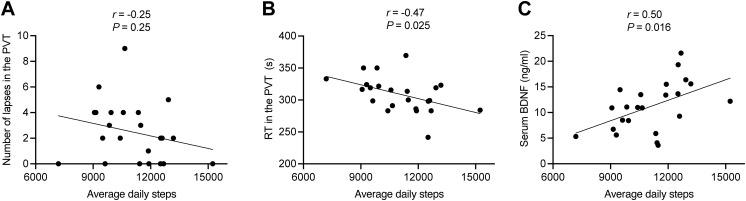
**(A, B)** The correlation between the average daily steps and the number of lapses **(A)** and RT **(B)** in the PVT. **(C)** The correlation between the average daily steps and serum BDNF level. RT, reaction time; PVT, Psychomotor vigilance task.

We found that increasing average daily steps were significantly associated with reduced RT in the PVT task (*r* = -0.47, *P* = 0.025) (**Figures 3A, B**). Additionally, the data demonstrated a positive relationship between average daily steps and blood BDNF levels (*r* = 0.50, *P* = 0.016) (**Figure 3C**). Our findings demonstrate that enhanced PA levels are associated with both improved sleep quality and attenuated next-day cognitive impairment, potentially mediated through upregulated BDNF production.

## Discussion

Our study systematically investigated the relationship between habitual PA and multidimensional sleep architecture among college students, incorporating both subjective and objective sleep assessments, alongside relevant physiological biomarkers and neurophysiological measurements (EEG power). Our results demonstrate that even moderate increments in average daily steps are associated with improvements in objective sleep parameters, particularly nocturnal SE and N2 stage duration, as well as reductions in wake stage and WASO. In parallel, greater PA predicted higher cognitive function and BDNF levels, and enhanced sleep EEG spectral features-findings that jointly support the hypothesis that PA may promote sleep-driven restoration and next-day functional capacity through both biological and neurophysiological pathways.

Sleep is a fundamental biological process, essential for cognitive health, emotional regulation, and overall somatic restoration (27, 28). The growing prevalence of insufficient or poor sleep quality-especially among young adults frequently engaged in sedentary or irregular lifestyles-underscores the need for easily implementable, non-pharmacological interventions (29). PA is widely recognized as one of the most robust, accessible health behaviors for promoting better sleep. Previous studies have highlighted that interventional exercise programs-including yoga, tai chi, and aerobic exercise-can ameliorate insomnia symptoms and improve both subjective and objective sleep quality by shortening SOL, enhancing sleep continuity, increasing TST, and minimizing nocturnal awakenings (30–32).

Differentiating itself from traditional interventional studies, our research systematically analyzed the real-world habitual PA-sleep architecture relationship. By leveraging continuous, objective ACT over a 7-day window paired with gold-standard PSG, we found that, among college students, higher average daily steps not only improved total nocturnal SE but also significantly increased N2 stage while shrinking WASO and wake stage. Multivariate regression further supported that daily steps positively predicted nocturnal SE and N2 time, and negatively predicted wake time. These findings corroborate and extend earlier evidence from intervention studies to more ecologically valid, everyday context among young adults. Given that N2 sleep comprises 50-60% of total sleep time across the night and serves as a crucial transitional stage from light (N1) to deeper sleep, its promotion is particularly relevant for the consolidation of cognitive and emotional functions (33–35). In our study, increased habitual PA was robustly correlated with greater N2 duration, underscoring the value of even modest increases in routine activity for optimizing restorative sleep microstructure. It is well-established that poor sleep often contributes to diminished daytime function, including impaired mood, cognitive processing, memory, and psychomotor vigilance (36). Conversely, improvements in nocturnal sleep quality can generate notable benefits for next-day performance. Our findings-demonstrating that more daily steps are linked to not only better sleep at night but also improved next-day psychomotor vigilance-strengthen this bidirectional framework (37). Research has proposed that the neurorestorative and memory-consolidating aspects of sleep depend critically on the quality and continuity of specific sleep stages, with N2 being prominent among them. Furthermore, our correlational analysis revealed that increased PA was associated with amplified power in EEG theta and sigma bands during NREM and REM sleep; these neuroelectrical patterns are closely tied to sleep stability, memory reactivation, and synaptic remodeling (38–40). Thus, daily PA, by promoting more coherent and restorative nighttime sleep, may help set the stage for better daytime alertness, learning, and productivity.

Despite the consistent association between increased PA and improved sleep, the precise underlying mechanisms remain multifactorial and incompletely understood. PA increases cerebral metabolic demand, resulting in the accumulation of sleep-promoting substances (notably adenosine) in the brain. Elevated adenosine facilitates sleep initiation and depth-its levels rise after exercise and decrease following recovery sleep, indicating its pivotal role in homeostatic sleep pressure (41). Exercise-induced elevations in core body temperature are followed by physiological cooling at sleep onset, which has been shown to promote deeper slow-wave sleep (42). Concurrently, habitual PA fosters parasympathetic dominance during sleep, particularly prominent in the second NREM cycle, reducing heart rate and supporting nocturnal autonomic stability (33). Research demonstrates that regular PA significantly elevates circulating BDNF levels, a key neurotrophin regulating neurogenesis, synaptic plasticity, and neural resilience (43). Mechanistically, exercise-induced lactate surges activate downstream CREB transcription factors through upregulated intracellular Ca^2+^ concentrations, thereby promoting BDNF biosynthesis; concurrently, enhanced neuroelectrical activity in prefrontal-hippocampal networks stimulates BDNF secretion via TrkB receptor-mediated PI3K/Akt and MAPK/ERK signaling phosphorylation cascades (44, 45). Our study’s observation that higher daily steps correlate with elevated serum BDNF supports the argument that PA-driven trophic support at the neurochemical level may underlie some of the sleep and cognitive benefits observed. Enhanced BDNF has been directly linked with better learning and cognitive performance, likely by supporting hippocampal circuitry engaged during sleep-dependent memory consolidation (46). The association we report between greater PA and enhanced NREM theta/sigma and REM theta power is notable. The sigma frequency band during NREM sleep corresponds to sleep spindles-hallmark electrophysiological features of NREM sleep that mediate memory consolidation and sleep stability maintenance (47, 48). Long-term exercise promotes increased spindle density and incidence, manifesting as elevated sigma power spectral density; concurrently, theta oscillations during REM sleep facilitate emotional memory processing and cognitive integration (49). Exercise-induced augmentation of REM sleep duration and intensity further elevates theta band power, potentially through enhanced neurometabolic activity involving lactate-mediated signaling cascades (50, 51). Thus, our data suggest that PA may serve as a modulator of brain activity patterns that both reflect and reinforce sleep’s cognitive and restorative functions. Bringing these lines of evidence together, we propose a plausible mechanistic model whereby increased PA during the day triggers favorable metabolic, neurotrophic, and neurophysiological changes, which in turn bolster sleep quality. Improved sleep, most notably via N2 and stabilized sleep microstructure, then enables better daytime cognitive and emotional functioning, potentially closing a virtuous feedback loop between waking activity, sleep, and neurobehavioral health.

An important yet frequently underappreciated issue highlighted by our study is the potential discrepancy between subjective and objective evaluations of sleep. In our study, sleep diaries did not consistently align with ACT or PSG findings (particularly regarding SOL and fragmentation), echoing concerns raised in previous literature (52). The discrepancy between subjective and objective sleep measures may arise from altered glucose metabolism in sensory processing and consciousness-related cortical regions, or from pathological high-frequency beta wave activity during NREM sleep inducing hallucinations of wakefulness (53). Furthermore, this psychophysiological dissociation often signals underlying psychological comorbidities, including anxiety, depression, and neuroticism, necessitating individualized clinical profiling based on patient-specific traits (54). Advancing the understanding of these mechanisms requires refined electrophysiological assessment methodologies, such as high-density EEG or combined neuroimaging approaches. Such discrepancies underscore the limitations of self-reported measures, which may be influenced by bias, inaccurate recall, or sleep misperception, and highlight the necessity for multimodal, objective data collection in both clinical and research contexts (55, 56). While our study offers novel, multi-layered insights into the link between PA, sleep quality, and neurobiological processes in a real-world cohort of young adults, certain limitations merit consideration. First, the study’s limited sample size and homogeneity in participant age and background constrain the generalizability of findings. Future investigations should incorporate middle-aged and elderly cohorts to comprehensively evaluate the effects of PA on sleep architecture and cognitive function. Second, we did not stratify types or intensities of PA beyond steps, nor did we capture long-term activity patterns or specific exercise regimens; future work should explore whether different modes or durations of PA differentially impact sleep architecture and next-day functioning. Third, the design was observational and correlative; longitudinal and interventional approaches will be essential to establish causality and to better characterize the temporal sequence of PA, sleep improvement, and daytime benefit. Fourth, while we identified BDNF and EEG power as putative mediators, direct experimental manipulations or mechanistic animal studies are needed to more definitively map the underlying pathways.

Nevertheless, our results suggest that even moderate increases in daily activity-readily achievable by most college students through lifestyle modifications or minimal behavioral interventions-can yield salutary effects on sleep and subsequent daytime cognitive function. In populations at risk for sleep complaints but not meeting diagnostic thresholds for insomnia disorder, PA emerges as an attractive, low-risk preventive and therapeutic strategy (57). Our findings indicate that increasing habitual PA, even at moderate levels, is associated with meaningful enhancements in nocturnal sleep quality among college students. Improvements are especially prominent in SE and N2 duration, accompanied by modulation of BDNF and EEG spectral markers, and better next-day psychomotor vigilance. These results provide experimental and theoretical foundation for incorporating PA as a practical, non-pharmacological adjunct in the management of poor sleep and its daytime consequences. Moreover, these research results may suggest that BDNF and the optimization of sleep neurophysiology may serve as key mechanistic bridges linking daytime activity, sleep, cognitive function, and next-day function (58, 59). Future research should expand sample diversity and size, dissect PA modalities and intensities, employ randomized controlled trials designs and integrate multi-omics and neuroimaging approaches to fully unravel the causal chains and molecular substrates involved. Ultimately, these efforts will not only inform the prevention and treatment of sleep problems in vulnerable populations, but also advance our understanding of the dynamic, bi-directional interplay between active waking, restorative sleep, and optimal daytime functioning.

## Data Availability

The original contributions presented in the study are included in the article/supplementary material. Further inquiries can be directed to the corresponding authors.
